# Comprehensive Evaluation and Spatial Difference Analysis of Regional Ecological Carrying Capacity: A Case Study of the Yangtze River Urban Agglomeration

**DOI:** 10.3390/ijerph16183499

**Published:** 2019-09-19

**Authors:** Yuanyuan Wang, Benhong Peng, Guo Wei, Ehsan Elahi

**Affiliations:** 1 School of Management Science and Engineering, Nanjing University of Information Science & Technology, Nanjing 210044, China; 2 Binjiang College, Nanjing University of Information Engineering, Wuxi 214015, China; 3 Department of Mathematics and Computer Science, University of North Carolina at Pembroke, Pembroke, NC 28372, USA; guo.wei@uncp.edu; 4 School of Business, Nanjing University of Information Science and Technology, Nanjing 210044, China; ehsanelahi@nuist.edu.cn

**Keywords:** Yangtze River urban agglomeration, ecological carrying capacity, ecological carrying elastic force, ecological carrying pressure, spatial difference

## Abstract

Ecological carrying capacity is an important factor of sustainable development for cities, and a critical part of achieving the coordinated development of the social economic and ecological environment for urban agglomerations. In order to evaluate the regional ecological carrying capacity and provide a basis for decision-making for regional sustainable development, this paper constructs an ecological carrying capacity model for the urban agglomeration from two dimensions: ecological carrying elastic force and ecological carrying pressure. The analytic hierarchy process is utilized to determine the weights of nine indices in these two dimensions. For the Yangtze River urban agglomeration, the comprehensive index of its ecological carrying capacity is investigated quantitatively, and the spatial distribution map of its comprehensive index measuring ecological carrying capacity is computed. The results show that Nanjing, Yangzhou, Taizhou, and Changzhou are in the stage of high load carrying; Suzhou, Wuxi, Nantong, and Zhenjiang are in the stage of low load carrying. In addition, the environmental protection investment has the greatest impact on ecological carrying elastic force, followed by the proportion of the tertiary industry; wastewater discharge has the greatest impact on ecological carrying pressure. The level of ecological carrying capacity varies within the region. It is necessary to take measures to increase the ecological carrying elastic force and reduce the ecological carrying pressure according to the actual conditions in each region. Meanwhile, exchanges and cooperation between different regions should be strengthened to stimulate the coordinated and sustainable development.

## 1. Introduction

In recent years, China’s economy has rapidly developed, and the level of urbanization has continuously upgraded. By the end of 2017, the urbanization rate of China’s permanent residents was 58.52%, increasing 40.6 percentage points from 1978, with an annual average increase of one percent [1]. This means that more and more rural people have moved to cities, increasing the occupation of various urban resources, which undoubtedly brings enormous ecological pressure on the development of cities and even the whole region. Based on the needs of the harmonious and green development of the social economy, the scientific assessment of regional ecological carrying capacity is becoming increasingly urgent.

The urban agglomeration is an important feature in the region. At present, taking urban agglomeration as the main body to stimulate urbanization has become another form of economic development. The ecological conditions of cities in urban agglomerations are different from each other, and studying the differences in the ecological carrying capacity for urban agglomerations is conducive to advancing its development quality as a whole, promoting its integrated development and ultimately boosting its regional ecological security. On the other hand, research on urban ecological carrying capacity by domestic and foreign scholars is increasingly enriched. 

In terms of the evaluation index of ecological carrying capacity, Li and Ma [2] (2013) selected indexes from the aspects of the economic environment, ecological protection, and environmental development to evaluate the ecological carrying capacity of Liaoning province. Zhang et al. [3] (2018) selected an evaluation index system with 18 indicators from the aspects of land carrying capacity and atmospheric environmental carrying capacity when studying China’s environmental and ecological carrying capacity. Xiong et al. [4] (2013) analyzed the spatial difference of Dongting Lake by selecting the resource and environment carrying index including wastewater discharge, energy consumption per unit added value, and socio-economic indicators such as population density, GDP per capita, etc. Furthermore, Li and Zhou [5] (2011), Du et al. [6] (2011), Jiang and Li [7] (2015), and Jia et al. [8] (2018) respectively studied the comprehensive evaluation index system of ecological carrying capacity in Changping District Pusalu Village, Jining city, Lihuagou Valley Region, and the water environment in China. Tang et al. [9] (2012) and Tang et al. [10] (2012) respectively investigated the evaluation index system and the policy choices of low-carbon manufacturing industry. Most scholars’ research on ecological carrying capacity is concentrated at the regional level, and the selection of evaluation indicators is also difficult to unify due to differences in research areas.

In terms of research methods on ecological carrying capacity, Chen et al. [11] (2011), Wu [12] (2014),Gao and Xu [13] (2014), and Miao et al. [14] (2016) respectively studied the ecological carrying capacity of Yangtze River Delta, Changsha, Jinlin province and Anhui Province, by using the ecological footprint method. Wang et al. [15] (2018) established a three-dimensional evaluation model for evaluating the carrying capacity of the regional ecological environment for social and economic development and carried out ecological research on 11 prefecture-level cities in Jiangxi Province. Wu et al. [16] (2018) studied urban rainstorm and waterlogging disasters based on microblogging data and the location-routing problem model of urban emergency logistics. Eric and Carrie [17] (2018) explored the impact of ecosystem levels on “carrying capacity” and examined mechanisms for controlling ecosystem carrying capacity. Peng et al. [18] (2016), taking the Dali Bai Autonomous Prefecture of Yunnan Province as an example, studied the evaluation method of ecological carrying capacity, combining human and nature, which provided a way to evaluate the development potential of mountainous cities. Tang et al. [19] (2015) and Yue et al. [20] (2013) used the state space method to evaluate the ecological carrying capacity of the Beijing-Tianjin-Hebei region and urban agglomeration in Liaoning Province, respectively. Yang and Hu [21] (2018) used the least squares regression method to study the spatial difference of ecological carrying capacity in Northern Shaanxi. Peng et al. [22] (2018) explored the governance of electronic waste recycling based on the theory of social capital embeddedness to discuss the proposal of improving ecological carrying capacity. At present, the research methods of ecological carrying capacity are various, but one common feature is that most of the methods are comprised of quantitative research, which is more concrete and intuitive. 

In terms of the evaluation model for ecological carrying capacity, Li et al. [23] (2011) constructed an evaluation model of ecological carrying capacity of shallow mountainous areas based on the study of ecosystem structure and carrying capacity and took Beijing’s shallow mountainous areas as an example to conduct an empirical study. Li et al. [24] (2011) established a comprehensive evaluation model of ecological carrying capacity, carried out in-depth analysis on its index system, weight definition, and data acquisition, and provided research ideas for the evaluation of ecological carrying capacity. Li et al. [25] (2017) constructed an ecological carrying pressure system and an ecological carrying elastic system model to analyze the ecological carrying capacity of Anhui Province comprehensively. Wang and Xu [26] (2015), Tian and Gang [27] (2012), Zhang et al. [28] (2012), and Zhang et al. [29] (2016) used the Pressure-State-Response (PSR) model to evaluate comprehensively the ecological carrying capacity of the water environment, Qinhuangdao city, Tianjin, and the lake ecosystem, respectively. Xu et al. [30] (2010) proposed an evaluation method of urban relative carrying capacity based on the grey relevant degree and applied it to Tongzhou district of Beijing. Wei et al. [31] (2017) and Zhou and Zhou [32] (2017) used the system dynamics model to measure the ecological carrying capacity of Beijing and the atmospheric environment in Wuhan City, respectively. Ma et al. [33] (2017) used the Dongtou Islands in China as an example to develop a system of indicators, which provided an application of the conceptual model in ECC (Ecological carrying capacity) evaluation. It can be seen that at present, the evaluation model of ecological carrying capacity has multiple different dimensions, and the index system is also different, which is still developing and improving.

In summary, domestic and foreign scholars pay more attention to the study of ecological carrying capacity, but there are two shortcomings: (1) The systematic nature of the evaluation index system for regional ecological carrying capacity has not been well studied in depth. The selection of evaluation indicators and the methods for determining the weight of indicators vary also. In particular, since the selection of indicators needs to be based on the actual socio-economic development of urban agglomerations, the scientific nature of the indicator system for urban agglomerations needs to be explored. (2) In terms of the evaluation method of ecological carrying capacity, most evaluation methods are not comprehensive, and there is a lack of integrated evaluation from the perspectives of the ecology, resources, environment, society, and economy.

Therefore, this paper focuses on constructing a comprehensive evaluation index system for the ecological carrying capacity for the Yangtze River urban agglomeration from two dimensions: ecological carrying elastic force and ecological carrying pressure, and utilizes the analytic hierarchy process to determine the index weight according to the connection between the indicators. Moreover, the spatial distribution of the ecological carrying capacity for Yangtze River urban agglomeration is analyzed. The research aims at exploring the comprehensive evaluation method of regional ecological carrying capacity, so as to provide effective suggestions for realizing regional sustainable development. 

In the remainder of the paper, the content is structured as follows. Section 2 introduces the research method of this paper, including the construction of the comprehensive evaluation index system of the ecological carrying capacity for the Yangtze River urban agglomeration, the determination of the index weight, and the construction of the urban ecological carrying capacity model from the aspects of ecological carrying elastic force and ecological carrying pressure. Section 3 investigates the problem of obtaining results, along with the analysis and discussion of the results. The ecological carrying capacity of Yangtze River urban agglomeration is comprehensively analyzed from the three dimensions of the ecological carrying elastic index, ecological carrying pressure index, and comprehensive index of ecological carrying capacity. Section 4 draws the conclusion and prospects and raises some questions to inspire future studies. 

## 2. Methodology

### 2.1. Study Area

With the issuance of “Opinions on accelerating the construction of Yangtze river urban agglomeration” by the Jiangsu provincial party committee and provincial government and the implementation of the “Development plan of Yangtze river urban agglomeration”, the Yangtze River urban agglomeration was born.

**Figure 1:**
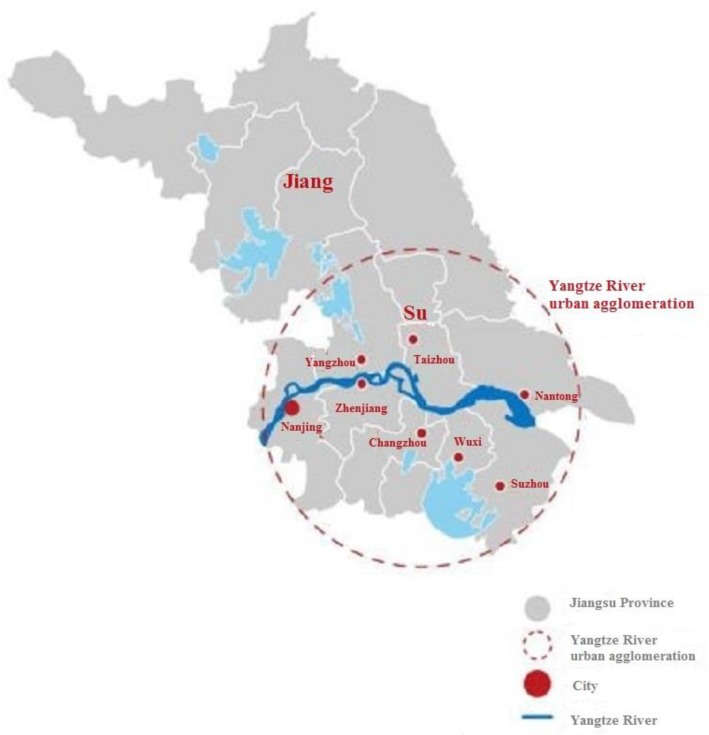
Map of the Yangtze River urban agglomeration.

The Yangtze River urban agglomeration is located along the Yangtze River in Jiangsu Province, China, covering Nanjing, Zhenjiang, Changzhou, Wuxi, Suzhou, Yangzhou, Taizhou, and Nantong (Figure 1). With an area of 51,000 square kilometers, a population of nearly 50 million, an economic scale of 6 trillion yuan, and a per capita GDP of more than 120,000 yuan [34], it occupies a large proportion in both Jiangsu and the national economic system. 

However, its traditional industry occupies a dominant position in industrial structure, and its economic development and environmental protection are not coordinated due to its large population density, large resource occupation and irregular agricultural development. Assessing the ecological carrying capacity of this region has become an important issue that cannot be ignored for sustainable development.

### 2.2. Construction of the Evaluation Index System of the Ecological Carrying Capacity for the Yangtze River Urban Agglomeration 

We selected some relevant indicators from two aspects: the ecological carrying elastic force and ecological carrying pressure, which was motivated by the literature research and the findings of Li and Ma [2] (2013), Li et al. [25] (2017), Xu [35] (2017), and Li et al. [24] (2011), in selecting the evaluation index of ecological carrying capacity, combined with the economic and ecological development status of the Yangtze River urban agglomeration, as well as by incorporating the framework of the ecological carrying capacity evaluation model.

This index system covers several aspects of the development of the Yangtze River urban agglomeration, including the social economy, population, industrial structure, environmental pollution, resource consumption, etc., which reflects the concept of sustainable development and the comprehensive evaluation of the whole index system.

The specific evaluation index system is shown in Table 1.

### 2.3. Construction of the Urban Ecological Carrying Capacity Model

Constructing a reasonable evaluation model of ecological carrying capacity is the premise and basis for the evaluation of ecological carrying capacity. In this paper, the ecological carrying capacity system is divided into the ecological carrying elastic system and the ecological carrying pressure system, so as to construct a comprehensive index model for the ecological carrying elastic force and ecological carrying pressure. Among them, we define ecological carrying capacity as the comprehensive reflection of ecosystem service capacity and social and economic development pressure; ecological carrying elastic force as the self-repairing ability of the ecological environment system; ecological carrying pressure as the direct negative impact of human activities on the natural ecological environment.

The urban ecological carrying elastic index is [2]:

(1) $$S=\overset{m}{\underset{j=1}{\sum }}X_{j}\times W_{j}$$

where $X_{j}$ represents the standardized value of the index layer of the elastic system and $W_{j}$ stands for the weight obtained from the index layer of the elastic system, j=1,2,⋯m,m=4.

The urban ecological carrying pressure index is [2]:

(2) $$P=\overset{n}{\underset{j=1}{\sum }}Y_{j}\times W_{j}$$

where $Y_{j}$ denotes the standardized value of the index layer of the pressure system and $W_{j}$ is the weight obtained by the index layer of the pressure system, j=1,2,⋯n, n=5.

The comprehensive index of urban ecological carrying capacity is [2]:

(3) D=P/S

where P represents the urban ecological carrying pressure index and S is the urban ecological carrying elastic index.

The combination of the ecological carrying pressure index with the ecological carrying elastic index can reflect the ecological carrying status of a city. Among them, the value of the ecological carrying elastic index reflects the level of urban ecological carrying capacity; the value of the ecological carrying pressure index reflects the level of urban ecological environment carrying pressure. The higher the pressure, the higher the comprehensive index of ecological carrying capacity. If the comprehensive index of ecological carrying capacity of a city is high, indicating that the urban ecosystem is in a stage of high load, the lower the ecological carrying capacity. 

The comprehensive index of urban ecological carrying capacity reflects the overall carrying status of a city.

### 2.4. Classification Criteria of the Urban Ecological Carrying Capacity

According to the research of Li et al. [25] (2017) and Xu [35] (2017), we determined the general classification criteria of urban ecological carrying capacity. See Table 2 for detailed classification standards. 

### 2.5. Analytic Hierarchy Process

At present, various methods are used to determine factor weights, including the analytic hierarchy process, principal component analysis, and the entropy method. Considering the fuzzy connection between the selected indicators and the complexity of the selected indicators in our study, we used the analytic hierarchy process to determine the index weights. The main steps are as follows [36]:

Firstly, the hierarchical model is established; secondly, a judgment matrix is established, in which the scale method of 1–9 is utilized, and the value is assigned according to its importance; thirdly, we calculate the weight vector, perform the consistency test, and calculate the weight value. In order to avoid the interference of other factors on the judgment matrix, the judgment matrix is required to meet the general consistency in practice, and the consistency test should be conducted according to Formula (4).

(4) CR=CI/RI

(5) $$\mathrm{CI}=\left(\lambda_{\max}-n\right)/\left(n-1\right)$$

where *CI* is the consistency index, *RI* the random consistency index, $\lambda_{\max}$ the largest characteristic root of the judgment matrix, and *n* the number of characteristic roots of the judgment matrix. when CR < 1.0, the judgment matrix is considered to be consistent, and the smaller the value of CR, the better the consistency of the judgment matrix. When CR ≥ 1.0, it is considered that the judgment matrix does not conform to the consistency and needs to be revised again. 

### 2.6. Data Source and Preprocessing

The data in this paper were selected from the 2017 statistical yearbook of Jiangsu province, the Environmental Statistical Bulletin of Jiangsu province, and the Statistical Yearbook and Environmental Statistical Bulletin of all cities in the Yangtze River urban agglomeration in 2017. 

Due to the differences in the measurement units, the nature and order of magnitude among the indicators in the indicator system, a unified dimensionless treatment was needed to eliminate the differences among the indicators. The specific method is as follows:

Standardized treatment formula of the benefit index:

(6) $$Z_{ij}=\left(x_{ij}-min\left\{x_{j}\right\}\right)/\left(max\left\{x_{j}\right\}-min\left\{x_{j}\right\}\right)$$

Standardized treatment formula of the cost index:

(7) $$Z_{ij}=\left(max\left\{x_{j}\right\}-x_{ij}\right)/\left(max\left\{x_{j}\right\}-min\left\{x_{j}\right\}\right)$$

where $Z_{ij}$ represents the value after standardization, $x_{ij}$ denotes the data of the jth evaluation index of city i, and $\max\left\{x_{j}\right\}$ and $\min\left\{x_{j}\right\}$ stand for the maximum and minimum values of the jth indicator in all cities. 

## 3. Analysis and Discussion of Results

### 3.1. Determination of the Weights of Indicators 

Through the judgment matrix of the analytic hierarchy process, Ten experts from government agencies, universities, and enterprises engaged in ecological and environmental research were invited to score the relative importance of the indices in the criterion Layer B and the indicator Layer C to calculate the index weights of the indicator layer. Then, the weighted average was utilized to summarize the scores of ten experts, so as to get the judgment matrix. The final results are shown in Table 3. 

It can be seen from Table 3 that C4 accounted for the largest proportion in the ecological carrying elastic system, indicating that the environmental protection investment had the greatest impact on ecological carrying capacity; C3 had the second largest proportion, implying that the proportion of the tertiary industry had a great impact on the ecological carrying elastic force. In the Yangtze River urban agglomeration, for cities with weak ecological carrying capacities, the ecological carrying capacity can be improved by increasing the environmental protection investment and adjusting the industrial structure.

In the ecological carrying pressure system, C6 accounted for the largest proportion, indicating that industrial wastewater discharge had the greatest impact on the ecological carrying pressure, followed by C9, industrial tailpipe emission. With the development of the city, the population has been expanding, and the discharge of wastewater and waste gas has also been increasing, which has caused tremendous pressure on the ecological environment. 

### 3.2. Analysis of Ecological Carrying Elastic Indices by Cities

**Figure 2:**
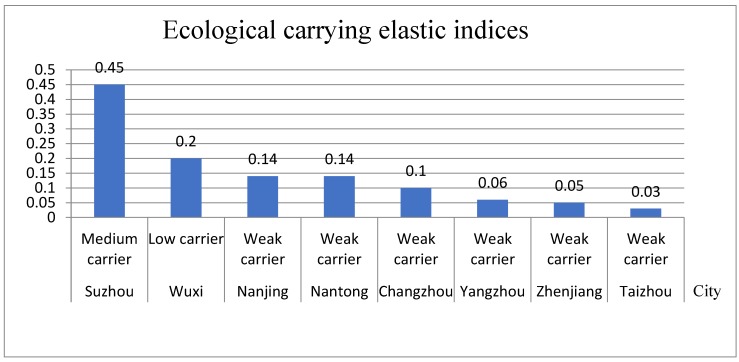
Histogram of ecological carrying elastic indices for the Yangtze River urban agglomeration.

Based on the ecological carrying elastic index model, the ecological carrying elastic indices by city were calculated. Then, the histogram of the ecological carrying elastic indices (Figure 2) can be obtained by combining with the grading evaluation standard of urban ecological carrying capacity.

It follows from Figure 2 that:

(1) Suzhou has the highest ecological carrying elastic index, which belongs to the medium carrying level. Due to the high level of urbanization in Suzhou, the proportion of the tertiary industry is relatively high, and the industrial structure is relatively perfect. At the same time, the environmental protection investment in Suzhou is higher, and the environmental protection is also stronger.

(2) Wuxi’s ecological carrying elastic index ranks the second, which belongs to a low carrying level. It has a relatively developed economy and a complete industrial structure, but its environmental investment is relatively lower when compared with Suzhou.

(3) The ecological carrying elastic indices for Nanjing, Nantong, and Changzhou are similar, at a weak carrying level. However, the reasons are slightly different. Nanjing and Changzhou have higher economic development levels and better industrial structures, so their ecological carrying elastic indices are relatively higher. Nantong has more investment in environmental protection, which partly compensates for the impact of the low economic development level and industrial structure imbalance on the ecological carrying elastic index. The ecological carrying capacities of Zhenjiang, Yangzhou, and Taizhou are also weaker and lag behind. Zhenjiang city, due to its low investment in environmental protection, has a lower ecological carrying elastic force; The economic development levels of Yangzhou and Taizhou are lower, and their industrial structures are not balanced, resulting in low levels of ecological carrying elastic force. 

In general, the ecological carrying elastic force of most cities in Yangtze River urban agglomeration is not high, at a weak carrying level, implying that the ecological carrying capacities of most cities are not high, and the environmental conditions are not optimistic. It is necessary to improve their ecological carrying capacities according to the specific conditions of each city.

### 3.3. Analysis of Ecological Carrying Pressure Indices by Cities

**Figure 3:**
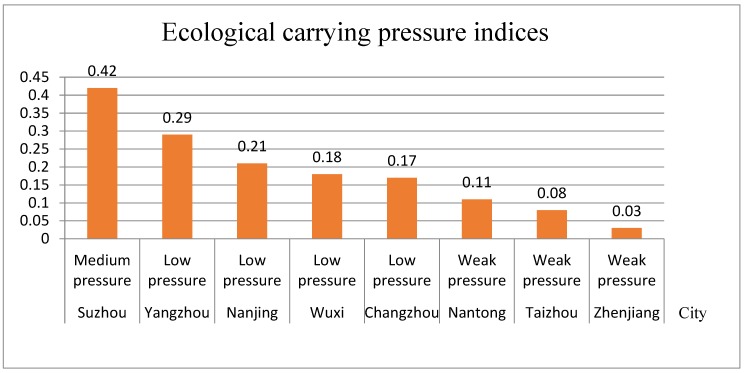
Histogram of ecological carrying pressure indices for Yangtze River urban agglomeration.

Through the ecological carrying pressure index model, the ecological carrying pressure indices of each city were calculated. Combined with the urban ecological carrying capacity grading evaluation criteria (Table 2), a histogram of the ecological carrying pressure indices was obtained (Figure 3).

From Figure 3, we can see that:

(1) Suzhou has the highest ecological carrying pressure index, which belongs to the medium pressure level. Suzhou’s industry has developed. Meanwhile, the population density is high, and hence, the exhaust emissions are large, while the occupancy of resources is large, and hence, the pressure on the ecological environment is relatively large.

(2) Nanjing, Changzhou, Yangzhou, and Wuxi are at low pressure levels, but the pressures are still high in Yangtze River urban agglomeration. The reason lies in their high population density and wastewater discharge. Among them, Yangzhou and Changzhou have increased their ecological carrying capacities to some extent due to the large amount of chemical fertilizers and pesticides applied.

(3) Taizhou, Zhenjiang, and Nantong are under weak pressure. Primarily due to the low discharge of wastewater and the small population pressure, the ecological carrying pressure is relatively small.

On the whole, the higher the level of economic development, the higher the corresponding ecological carrying pressure. At the same time, most of the cities in the Yangtze River urban agglomeration have low ecological carrying pressure, and most of them are in a stage of low carrying pressure or weak carrying pressure. However, with the further development of the Yangtze River urban agglomeration, the ecological carrying pressure will increase, and measures still need to be taken to decrease the ecological carrying pressure of each city. Especially in Suzhou, the economic development and population density lead to the large amount of resources and the maximum ecological carrying pressure.

### 3.4. Analysis of the Comprehensive Indices of the Ecological Carrying Capacity

Through the comprehensive index model of urban ecological carrying capacity, with the ecological carrying elastic indices (Figure 2) and ecological carrying pressure indices (Figure 3) in the Yangtze River urban agglomeration, combined with the urban ecological carrying capacity grading evaluation standard (Table 2), the comprehensive indices and ranking table of ecological carrying capacity in Yangtze River urban agglomeration were obtained (Table 4).

From Table 4 we can see that:

(1) Nanjing, Changzhou, Taizhou, and Yangzhou are under high load. The economic development levels of Nanjing, Changzhou, Suzhou, and Wuxi are higher and the population density higher, and so are the waste and pollutant emissions of production and living. Therefore, the ecological carrying elastic indices and ecological carrying pressure indices of these four cities are higher than other cities. Among them, the ecological carrying elastic indices of Nanjing and Changzhou are lower than those of their ecological carrying pressure indices because the environmental protection investment cannot maintain their huge population and economic development. Taizhou and Yangzhou have a relatively low level of economic development, an underdeveloped industrial structure, a high use of chemical fertilizers and pesticides in agricultural development, and a low investment in environmental protection, resulting in huge ecological carrying pressure. Therefore, the comprehensive indices of their ecological carrying capacities are high, and they are in a stage of high load.

(2) Zhenjiang, Wuxi, Suzhou, and Nantong are under low load. Among them, Zhenjiang has a low comprehensive index of ecological carrying capacity due to low population density and low production and living emissions. Nantong has more environmental protection investment, and the ecological carrying elastic index is higher than the ecological carrying pressure index, so the comprehensive index of ecological carrying capacity is low. Although Suzhou and Wuxi have large emissions from production and living, they have a large investment in environmental protection. Therefore, they are in a low-load stage. However, the ecological carrying pressure indices of Suzhou and Wuxi are slightly lower than the ecological carrying elastic indices, and the comprehensive indices of ecological carrying capacity are close to one, which means close to ecological balance. 

In short, the comprehensive indices of the ecological carrying capacity of Yangzhou, Taizhou, Changzhou, and Nanjing are higher in the Yangtze River urban agglomeration and are in the stage of high load carrying, indicating that the development of these cities has exceeded the capacity of the regional ecological environment and is in an unsustainable development state. These cities should pay more attention to environmental inputs while developing their economies. For Wuxi, Suzhou, Nantong, and Zhenjiang, although they are in a low-load stage, they still need to pay attention to environmental protection and reduce the comprehensive indices of ecological carrying capacity, especially Wuxi and Suzhou: their comprehensive indices of ecological carrying capacities were close to one.

**Figure 4:**
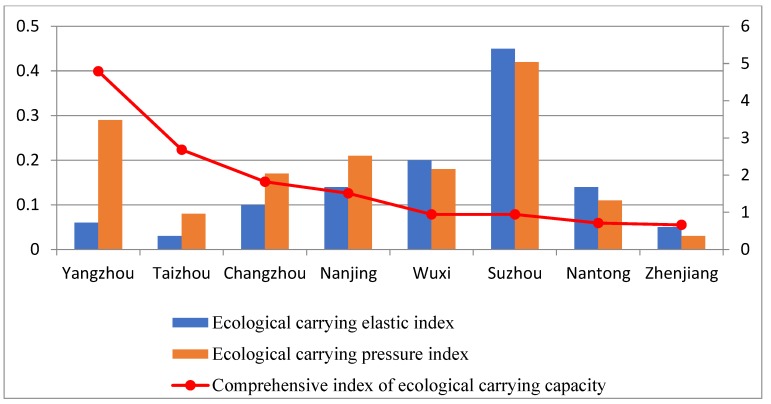
Comprehensive evaluation indices figure of the ecological carrying capacities for the Yangtze River urban agglomeration.

According to the ecological carrying elastic indices (Figure 2), the ecological carrying pressure indices (Figure 3), and the ecological carrying capacity comprehensive indices (Table 4), the comprehensive evaluation indices figure of the ecological carrying capacities for Yangtze River urban agglomeration was obtained (Figure 4).

Through further analysis, we found that environmental protection investment and industrial structure had the greatest impact on ecological carrying elastic force. The proportion of tertiary industry and the investment in environmental protection in Nanjing, Suzhou, Nantong, and Wuxi were higher than other cities, making their ecological carrying elastic indices higher than other cities. It can be seen that a reasonable industrial structure can enhance the ecological carrying capacity of the region; the environmental protection investment can strengthen the cycle mechanism of the ecosystem, thereby improving the ecological carrying elastic force. Suzhou’s environmental protection investment had a larger proportion, which improved its ecological carrying elastic force to a certain extent, therefore improving its comprehensive carrying capacity.

Secondly, the wastewater discharge had the greatest impact on ecological carrying pressure, higher for Suzhou, Wuxi, Nanjing, and Yangzhou than other cities, making their ecological carrying pressure indices higher. The use of chemical fertilizers and pesticides in agricultural production also exerted a certain pressure on the ecological environment, most significant for Changzhou and Yangzhou. The use of large amounts of chemical fertilizers and pesticides aggravated the regional ecological carrying pressure and had a large negative impact on the ecological environment.

### 3.5. Discussion of Results 

**Figure 5:**
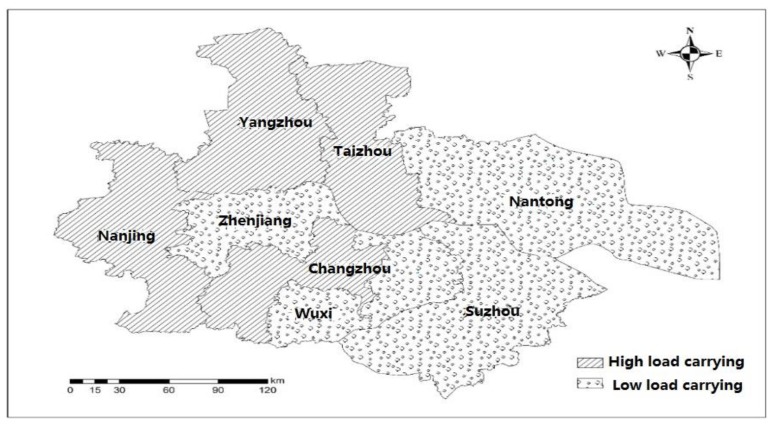
Spatial distribution map of the comprehensive indices of ecological carrying capacity by city.

By utilizing the comprehensive indices and ranking table of the ecological carrying capacity for the Yangtze River urban agglomeration (Table 4) and the grading evaluation standard of the urban ecological carrying capacity (Table 2), combined with the administrative region division map of the Yangtze River urban agglomeration, the ArcGIS software (Esri, Redlands, CA, USA) was used to produce the spatial distribution diagram of the ecological carrying capacity of eight cities in the Yangtze River urban agglomeration, as shown in Figure 5. 

Figure 5 shows that:

(1) Nanjing, Yangzhou, Taizhou, and Changzhou are under high load of carrying conditions. First of all, these cities are farther away from the estuary of the Yangtze River, and their own ecosystem circulation is weaker. Among them, Nanjing’s industrial structure is relatively complete, but the environmental protection investment is relatively low; meanwhile, as a political and cultural center of Jiangsu province, Nanjing is densely populated, resulting in high wastewater and gas emissions. For the economically-developed cities like Nanjing, we should pay more attention to environmental protection investments, optimize the population structure, and reduce the environmental pollution and resource waste. Yangzhou and Taizhou have lower economic development levels, imperfect industrial structure, and insufficient investment in environmental protection, and their comprehensive indices of ecological carrying capacity are higher due to the higher discharge of wastewater and waste gas and the heavier use of chemical fertilizers and pesticides. For such cities, local resources should be fully utilized to develop the economy and improve the industrial structure; at the same time, increasing investment in environmental protection, raising public awareness of environmental protection, and improving resource utilization. Changzhou’s industrial structure is improper; the agricultural production behavior is not standardized; and environmental protection investment is insufficient. For such cities, we should first focus on the transformation and upgrading of the industrial structure while developing the economy, and at the same time increase investment in environmental protection; second, increasing investment in science and technology, stimulating the transformation of the agricultural production mode, and reducing the use of fertilizers and pesticides.

(2) Suzhou, Wuxi, Nantong, and Zhenjiang are under a lower load of carrying conditions. Except Zhenjiang, other cities are close to the estuary of the Yangtze River, and their ecosystems have stronger circulation capacities. Like Suzhou, it is densely populated and has a large amount of resources, but the industrial structure is good and the environmental protection investment higher. Therefore, although having a higher load of carrying and higher pressure state, it has only a lower load. For such cities, it is still necessary to optimize the industrial structure and strengthen the formulation of relevant environmental policies. On the contrary, Zhenjiang has a better environmental condition; at the same time, wastewater discharge is lower, and it is a lowlier loaded, lower pressure state. For such cities, we should vigorously encourage the tertiary industry such as the service industry and tourism on the basis of making full use of the advantages of local natural resources and continuously increase environmental protection investments in the later stage of development. The economic development level of Nantong is a little lower; the proportion of tertiary industry is low; but its environmental investment is high, and population density is low. For such cities, it is necessary to develop the tertiary industry on the basis of geographical advantages and accelerate the transformation and upgrading of the industrial structure. Wuxi has a relatively high level of economic development and a relatively complete industrial structure. At the same time, due to the higher investment in environmental protection and less pollution in agricultural development, its ecological environment is good. For such cities, the industrial structure should be continuously optimized during the development process, and investment in environmental protection should be increased. On the other hand, we should also increase the investment in science and technology, improve resource utilization, and formulate relevant policies such as sewage tax to reduce pollutant emissions, thereby maintaining the low-load carrying stage.

## 4. Conclusions

The comprehensive evaluation index system of urban ecological carrying capacity is the basis for urban carrying capacity assessment and the focus of sustainable development. The assessment of the ecological carrying capacity for urban agglomerations is conducive to guiding the industrial structure and development direction for each city and has positive significance for promoting urbanization. We have established such a system for the Yangtze River urban agglomeration from two dimensions, ecological carrying elastic force and ecological carrying pressure, and analyzed the spatial difference of its ecological carrying capacity. The results revealed that:

(1) The scientific evaluation of ecological carrying capacity requires comprehensive evaluation from two dimensions: ecological carrying elastic force and ecological carrying pressure. The more developed areas in the region tended to have larger resource occupations and thus higher ecological carrying pressure; moreover, due to the relatively complete industrial structure and high technical level, the ecological carrying elastic force of these areas was also higher. In areas where the ecological carrying elastic index was lower than the ecological carrying pressure index, the ecosystem was in a higher load stage because it could not maintain the ecological pressure brought about by its production and life; the areas where the ecological carrying elastic index was higher than the ecological carrying pressure index had lower ecological pressure and were in the stage of lower load.

(2) Environmental protection investment had the greatest impact on ecological carrying elastic force, followed by the proportion of the tertiary industry; wastewater discharge had the greatest impact on ecological carrying pressure. For example, the proportion of tertiary industry and environmental protection investment in Nanjing, Suzhou, Nantong, and Wuxi were higher than other cities, and so were their ecological carrying elastic indices. Such densely-populated and economically-developed cities as Suzhou and Nanjing had larger relatively complete systems treating wastewater and waste gas, and therefore, the pressure on the ecological environment was naturally heavier.

(3) The ecological conditions of cities in the Yangtze River urban agglomeration were different. It is urgent to build an exchange and cooperation mechanism for urban agglomerations to improve the quality of the overall ecological environment. First of all, municipal governments should take measures to improve the ecological carrying elastic force and reduce the ecological carrying pressure according to the actual conditions of each city. For example, for cities with large problems in industrial structures and an insufficient proportion of tertiary industry like Yangzhou, Taizhou, Zhenjiang, and Nantong, the key is to stimulate the transformation and upgrading of their industrial structures. Secondly is strengthening communication and cooperation among cities in the development process and highlighting the leading role of such central cities as Suzhou, sharing green development experiences with cities for decision-making. Thirdly is setting up special supervision departments to strengthen the supervision and inspection of the ecological environment in urban agglomerations, such as strict control of wastewater and waste gas emissions, strengthening the supervision and guidance of agricultural practices. Finally is coordinating regional development and establishing a complete urban ecological economic system. The mutually beneficial urban ecological network is used to facilitate the coordinated development of ecology and economy among cities and between urban and rural areas.

The innovations of this paper are as follows: (i) most of the existing research is on typical provinces, cities, or regions, but there is a lack of research from the perspective of the urban agglomeration. The construction of the index system and the evaluation of ecological carrying capacity for the urban agglomeration in this paper enrich the research of ecological carrying capacity for urban agglomerations. (ii) Different from previous studies evaluating ecological carrying capacity from a single perspective, we established a comprehensive evaluation index system for the Yangtze River urban agglomeration from the two dimensions of ecological carrying elastic force and ecological carrying pressure, which is a combination of ecological and social economic perspectives. Furthermore, the advantage of this paper is that it is not limited to specific research conclusions. Through the comprehensive evaluation of ecological carrying capacity for the Yangtze River urban agglomeration, it points out the general standard for evaluating regional ecological carrying capacity, which is more universal. Meanwhile, the indicator system established in this study lays a foundation for the establishment of a more scientific indicator system in the future and also provides a decision-making basis for the sustainable development of urban agglomerations. 

However, due to the large amount of data required for the evaluation of the ecological carrying capacity of the ecological composite system in this study, the complicated process of obtaining the data, the limitation of creating indicators, and the subjectivity in determining the weight of the index, subsequent research for other areas with significant conditions needs to be conducted to further optimize the indicator system, the method of determining the index weight can also choose a more scientific method combining subjective and objective weighting, especially for the creation of indicators; for example, tailpipe emissions are derived from the conversion of various types of exhaust gases into standard conditions. However, some air pollutants may not be suitable for conversion to a standard state, or simply adding them together has little impact on research problems; follow-up research will carefully consider these issues, and the creation of indicators will be more rigorous. Then, the improved ecological carrying capacity evaluation model will have better scientific and universal value.

## Figures and Tables

**Table 1:** Comprehensive evaluation index system of ecological carrying capacity for the Yangtze River urban agglomeration.

Target Layer	Criteria Layer	Index Layer (Unit)	Source of Indicators
A1 Ecological carrying capacity	B1 Ecological carrying elastic force	C1 Urbanization level (%)	[2,25,35]
		C2 GDP per capita (yuan)	
		C3 Proportion of tertiary industry (%)	
		C4 Environmental investment (billion)	
	B2 Ecological carrying pressure	C5 Population density (person/km^2^)	
		C6 Industrial wastewater discharge (billion tons)	
		C7 Consumption of chemical fertilizers (tons)	
		C8 Consumption of chemical pesticides (tons)	
		C9 Industrial tailpipe emission (10,000/m^3^)	

Note: the urbanization level is also called the urbanization rate, which refers to the proportion of urban population to the total population; the tertiary industry refers to the industry that serves life and production, that is the service industry.

**Table 2:** Classification evaluation criteria for urban ecological carrying capacity.

Index Value	Elastic System	Pressure System	Index Value	Comprehensive Index of Carrying Capacity
0~0.15	Weak carrier	Weak pressure	D<1	Low load carrying capacity
0.16~0.30	Low carrier	Low pressure		
0.31~0.45	Medium carrier	Medium pressure	D=1	Carrying pressure balance
0.46~0.60	High carrier	High pressure		
0.61~0.75	Higher carrier	higher pressure	D>1	High load carrying capacity

**Table 3:** Weights of the comprehensive evaluation indices for ecological carrying capacity.

Criterion Layer	Indicator Layer	Weight
B1 Ecological carrying elastic force	C1 Urbanization level	0.0329
	C2 GDP per capita	0.0506
	C3 Proportion of tertiary industry	0.0864
	C4 Environmental investment	0.3301
B2 Ecological carrying pressure	C5 Population density	0.0213
	C6 Industrial wastewater discharge	0.2983
	C7 Consumption of chemical fertilizers	0.0546
	C8 Consumption of chemical pesticides	0.0369
	C9 Industrial tailpipe emission	0.0888

**Table 4:** Comprehensive indices and ranking table of ecological carrying capacity.

City	Comprehensive Indices of Ecological Carrying Capacity	Level	Order
Yangzhou	4.79	High load	1
Taizhou	2.68	High load	2
Changzhou	1.82	High load	3
Nanjing	1.51	High load	4
Suzhou	0.94	Low load	5
Wuxi	0.94	Low load	6
Nantong	0.71	Low load	7
Zhenjiang	0.66	Low load	8
